# Methylglyoxal as a new biomarker in patients with septic shock: an observational clinical study

**DOI:** 10.1186/s13054-014-0683-x

**Published:** 2014-12-12

**Authors:** Thorsten Brenner, Thomas Fleming, Florian Uhle, Stephan Silaff, Felix Schmitt, Eduardo Salgado, Alexis Ulrich, Stefan Zimmermann, Thomas Bruckner, Eike Martin, Angelika Bierhaus, Peter P Nawroth, Markus A Weigand, Stefan Hofer

**Affiliations:** Department of Anesthesiology, University of Heidelberg, 110, Im Neuenheimer Feld, D-69120 Heidelberg, Germany; Department of Medicine I and Clinical Chemistry, University of Heidelberg, 410, Im Neuenheimer Feld, D-69120 Heidelberg, Germany; Department of General and Transplant Surgery, University of Heidelberg, 110, Im Neuenheimer Feld, D-69120 Heidelberg, Germany; Department of Infectious Diseases, University of Heidelberg, 324, Im Neuenheimer Feld, D-69120 Heidelberg, Germany; Institute of Medical Biometry and Informatics, University of Heidelberg, 305, Im Neuenheimer Feld, D-69120 Heidelberg, Germany

## Abstract

**Introduction:**

The role of reactive carbonyl species, such as methylglyoxal (MG), has been overlooked within the context of the sepsis syndrome. The aims of this study were to assess the impact of MG formation in different inflammatory settings and to evaluate its use for early diagnosis as well as prognosis of the sepsis syndrome.

**Methods:**

In total, 120 patients in three groups were enrolled in this observational clinical pilot study. The three groups included patients with septic shock (n = 60), postoperative controls (n = 30), and healthy volunteers (n = 30). Plasma samples from patients with septic shock were collected at sepsis onset and after 24 hours and 4, 7, 14, and 28 days. Plasma samples from postoperative controls were collected prior to surgery, immediately following the end of the surgical procedure as well as 24 hours later and from healthy volunteers once. Plasma levels of MG were determined by high-performance liquid chromatography. Additionally, plasma levels of procalcitonin, C-reactive protein, soluble CD14 subtype, and interleukin-6 were determined.

**Results:**

Patients with septic shock showed significantly higher plasma levels of MG at all measured times, compared with postoperative controls. MG was found to identify patients with septic shock more effectively—area under the curve (AUC): 0.993—than procalcitonin (AUC: 0.844), C-reactive protein (AUC: 0.791), soluble CD14 subtype (AUC: 0.832), and interleukin-6 (AUC: 0.898) as assessed by receiver operating characteristic (ROC) analysis. Moreover, plasma levels of MG in non-survivors were significantly higher than in survivors (sepsis onset: *P = 0.018 for 90-day survival; **P = 0.008 for 28-day survival). Plasma levels of MG proved to be an early predictor for survival in patients with septic shock (sepsis onset: ROC-AUC 0.710 for 28-day survival; ROC-AUC 0.686 for 90-day survival).

**Conclusions:**

MG was identified as a marker for monitoring the onset, development, and remission of sepsis and was found to be more useful than routine diagnostic markers. Further studies are required to determine the extent of MG modification in sepsis and whether targeting this pathway could be therapeutically beneficial to the patient.

**Trial registration:**

German Clinical Trials Register DRKS00000505. Registered 8 November 2010.

**Electronic supplementary material:**

The online version of this article (doi:10.1186/s13054-014-0683-x) contains supplementary material, which is available to authorized users.

## Introduction

Sepsis represents an ongoing challenge in intensive care units (ICUs) and remains the most common cause of mortality in this setting [1,2]. Increased oxidative stress, as a consequence of the systemic inflammatory response, has been suggested as a major causative factor for the development and progression of the disease. However, there is little conclusive evidence that a targeted treatment with antioxidants in patients with sepsis could be beneficial [3,4]. This may suggest the involvement of alternative mediators of cellular stress in the pathophysiology of sepsis.

Reactive carbonyl species (RCS) have emerged as effective mediators of cellular dysfunction. RCS are a heterogeneous group of reactive low-molecular-weight carbonyls, which are able to interact with various biomolecules, such as proteins, deoxyribonucleic acid, or phospholipids, resulting in structural distortions and functional impairment [5]. The detrimental effects of RCS are comparable to those caused by reactive oxygen species (ROS), except that RCS are significantly more stable and can readily diffuse out of the cell and have effects far from the original site of their formation [5]. Furthermore, several of the most reactive RCS are derived from glucose metabolism (in particular, glycolysis) [5]. As ROS require three stages of metabolism before they are produced, RCS can be viewed as providing a more direct insult to the macromolecular integrity of the cell.

Methylglyoxal (MG) is a highly reactive RCS, produced endogenously from the spontaneous degradation of triosephosphates—glyceraldehyde-3-phosphate (GA3P) and dihydroxyacetone phosphate (DHAP)—during glycolysis. It is estimated that approximately 0.089% of triosephosphates are converted to MG and that the total body rate of formation for a healthy adult human is about 3 mmol per day [6]. The formation of MG is closely linked to the rate of glycolysis within the cell. It would be expected that under physiological conditions, where there is either an increase in glycolytic flux or an increased dependence on glycolysis for energy, the rate of MG formation would be increased [7,8]. This has proven to be the case in patients with diabetes mellitus, in which complications such as nephropathy, neuropathy, and retinopathy can be linked to increases in cellular levels of glycated proteins, which then are referred to as advanced glycation end products (AGEs) [9].

A clinical feature of patients with sepsis is hyperglycemia [10,11]. However, the consequences of elevated blood glucose in sepsis, particularly with respect to production of MG and its potential role in the development and progression of the disease, have yet to be fully investigated. The aims of this study were to assess the impact of MG formation in different inflammatory settings and to evaluate its use for early diagnosis as well as prognosis of sepsis.

## Materials and methods

### Selection of patients and study procedures

The observational clinical pilot study was approved by the local ethics committee (Ethics Committee of the Medical Faculty of Heidelberg: Trial Code Number S123-2009/German Clinical Trials Register: DRKS00000505) and was conducted in the surgical ICU of the University Hospital of Heidelberg, Germany. Study and control patients or their legal designees provided written informed consent. In total, 120 patients in three groups were consecutively enrolled into the study from August 2009 to July 2010. The three groups were the following: (1) 60 patients with septic shock, according to the criteria of the International Sepsis Definitions Conference [12] (referred to as the septic group, or S) (Table 1), due to documented or suspected infection according to the criteria of the International Sepsis Forum Consensus Conference on Definitions of Infection in the ICU (Additional file 1: Table S1) [13]; (2) 30 postoperative controls following major abdominal surgery without any evidence of infection (the postoperative group, or P) (Table 2); and (3) 30 healthy volunteers (the volunteer group, or V) (Table 2). Blood samples from patients with septic shock were collected at sepsis onset (T0) and 24 hours (T1), 4 days (T2), 7 days (T3), 14 days (T4), and 28 days (T5) later. Blood samples from the postoperative group were collected prior to surgery (T0), immediately following the end of the surgical procedure (T1), and 24 hours later (T2). Blood samples from the volunteer group were collected once (T0).

**Table 1:** **Characteristics of 60 patients in the septic group**

**Septic group (n = 60)**		
**Demographic data**		
Age, years	70 (64-76)	
Male sex	46 (76.7%)	
ASA status: I; II; III; IV; V	1 (1.7%); 11 (18.3%); 29 (48.3%); 15 (25.0%); 1 (1.7%)	
**Primary site of infection (double-naming feasible)**		
Lung	12 (20.0%)	
Gastrointestinal tract	32 (53.3%)	
Genitourinary tract	6 (10.0%)	
Others	18 (30.0%)	
Unknown	4 (6.7%)	
**Infection type**		
Gram-positive isolates	16 (26.7%)	
Gram-negative isolates	16 (26.7%)	
Combination of both	18 (30.0%)	
Suspected infection without any microbiological finding	10 (16.7%)	
**Septic organ failures**		
Septic shock	60 (100.0%)	
Acute renal failure	35 (58.3%)	
Acute respiratory distress syndrome	49 (81.2%)	
Acute liver failure	15 (25.0%)	
**Disease severity scoring**	**S/T0**	**S/T1**
APACHE II score	33 (27-38)	33 (28-39)
SAPS	75 (66-86)	73 (64-84)
SOFA score	14 (11-15)	14 (12-15)
**Clinical data**	**S/T0**	**S/T1**
Norepinephrine, μg/kg per minute	0.20 (0.06-0.31)	0.20 (0.08-0.32)
Maximum heart rate, 1/minute	115 (97-130)	113 (102-127)
Minimum MAP, mm Hg	57 (50-64)	60 (54-66)
FiO_2_, none	0.65 (0.50-0.80)	0.58 (0.46-0.70)

Data are presented as number (percentage) or as median (with quartiles). APACHE II, Acute Physiology and Chronic Health Evaluation II; ASA status, physical status classification system according to the American Society of Anesthesiologists; FiO_2_, fraction of inspired oxygen; MAP, mean arterial pressure; SAPS, Simplified Acute Physiology Score; SOFA, Sequential Organ Failure Assessment.

**Table 2:** **Characteristics of 30 patients in the postoperative group and 30 individuals in the volunteer group**

**Postoperative group (n = 30)**	
**Demographic data**	
Age, years	62 (57-70)
Male sex	16 (53.3%)
ASA status: I; II; III; IV; V	0 (0.0%); 9 (30.0%); 20 (66.7%); 1 (3.3%); 0 (0.0%)
**Site of surgery (double-naming feasible)**	
Liver	7 (23.3%)
Pancreas	11 (36.7%)
Gastrointestinal	27 (90.0%)
**Volunteer group (n = 30)**	
**Demographic data**	
Age, years	26 (24-28)
Male sex	19 (63.3%)
ASA status: I; II; III; IV; V	21 (70.0%); 9 (30.0%); 0 (0.0%); 0 (0.0%); 0 (0.0%)

Data are presented as number (percentage) or as median (with quartiles). ASA status, physical status classification system according to the American Society of Anesthesiologists.

### Immunoassays

Plasma concentrations of total antioxidant capacity (TAC), methylglyoxal-derived advanced glycation end products (MG-AGE), and interleukin-6 (IL-6) were measured by using enzyme-linked immunosorbent assay (ELISA) kits in accordance with the instructions of the manufacturer (TAC and MG-AGE: Biocat, Heidelberg, Germany; IL-6: R&D Systems, Minneapolis, MN, USA). Plasma levels of soluble CD14 subtype (sCD14-ST) were measured by using the Pathfast Presepsin chemiluminescent enzyme immunoassay in accordance with the instructions of the manufacturer (Mitsubishi Chemical, Tokyo, Japan).

### Methylglyoxal measurements with high-performance liquid chromatography

The concentrations of MG in plasma were determined by derivatization with 1,2-diamino-4,5-dimethoxybenzene and high-performance liquid chromatography of the quinoxaline adduct by fluorescence detection [14,15].

### Preparation of peripheral blood mononuclear cells

Peripheral blood mononuclear cells (PBMCs) were separated immediately after blood collection. Ethylenediaminetetraacetic acid (EDTA)-anticoagulated whole blood was loaded carefully onto a lymphocyte separation medium (PAA Laboratories GmbH, Pasching, Austria) and centrifuged for 25 minutes at 1,200 rpm without brakes at 4°C. The PBMC-containing band was aspirated, and the cells were washed three times with NaCl 0.9%.

### RNA extraction and quantitative polymerase chain reaction

RNA extraction was performed by using the column-based RNeasy Plus Mini Kit (Qiagen, Hilden, Germany) in accordance with the instructions of the manufacturer. RNA (250 ng) was reverse-transcribed by using the QuantiTect Reverse Transcription Kit (Qiagen) in accordance with the instructions of the manufacturer. Subsequent real-time polymerase chain reaction (PCR) analysis was performed on a StepOnePlus PCR cycler (Applied Biosystems, Weiterstadt, Germany) by using predesigned TaqMan assays for glyoxalase-1 (GLO-1) (assay ID Hs00198702_m1) and β-Actin (assay ID Hs99999903_m1). PCRs were set up by using the TaqMan Universal PCR Master Mix (Applied Biosystems, Weiterstadt, Germany). All experiments were run in triplicate. Results are interpreted by calculating the change in cycle threshold (∆Ct) value (Ct β-actin – Ct GLO-1) of each sample.

### Statistical analysis

The present clinical investigation was conducted as a pilot study. Group sizes were set at 60 individuals in the septic group and 30 individuals in both the healthy and postoperative groups. Resulting study data was entered into an electronic database (Microsoft Excel 2010, Microsoft Corporation, Redmond, WA, USA) and evaluated by using SPSS software (Statistical Product and Services Solutions, Version 20.0, SPSS Inc., Chicago, IL, USA) or Graphpad Prism for Macintosh (Version 5.0f, GraphPad Software, San Diego, CA, USA). Categorical data were summarized by means of absolute and relative frequencies. Quantitative data were summarized by using medians (with quartiles). Wherever appropriate, data was visualized by using line or bar charts. The Kolmogorov-Smirnov test was applied to check for normal distribution. Owing to non-normally distributed data in this study, non-parametric methods for evaluation were used. Furthermore, a receiver operating characteristic (ROC) curve was calculated with suitable parameters in order to create cutoff values to determine the diagnostic or prognostic value of each parameter with regard to the diagnosis as well as the prognosis of sepsis. Comparisons of the areas under two or more correlated ROC curves were performed as described by Delong *et al*. [16]. Correlation analysis was performed calculating Spearman-Rho (r). A *P* value of less than 0.05 was considered statistically significant. The following symbols were used with regard to higher orders of significance: *P <0.05, **P <0.01, ***P <0.001.

## Results

In total, 120 patients in three groups were subjected for evaluation. A detailed characterization of the different groups is presented in Tables 1 and 2.

### Kinetics of methylglyoxal**-**derived carbonyl stress in human sepsis

**Figure 1:**
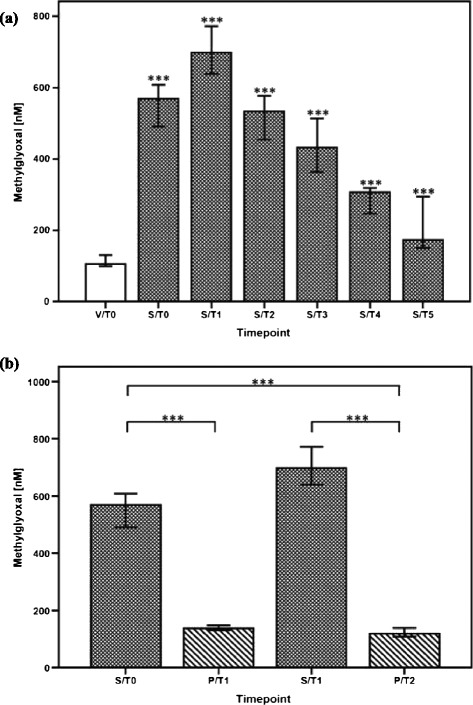
**Plasma levels of methylglyoxal in different inflammatory settings in humans. (a)** Comparison of plasma methylglyoxal measurements in healthy volunteers (n = 30, white bar) and patients with septic shock (n = 60, white-spotted bars in black color). Plasma levels of methylglyoxal in patients with septic shock are presented at sepsis onset (S/T0) and 24 hours (S/T1), 4 days (S/T2), 7 days (S/T3), 14 days (S/T4), and 28 days (S/T5). Plasma methylglyoxal measurements in healthy volunteers were performed once (V/T0). Data in bar charts are presented as medians and 95% confidence intervals (CIs). With regard to symbolism and higher orders of significance: ***P <0.001. Asterisks refer to pairwise comparisons between healthy volunteers and patients with septic shock at the presented time points. **(b)** Comparisons of plasma methylglyoxal measurements in postoperative controls following major abdominal surgery (n = 30, white-striped bars in black color) and patients with septic shock (n = 60, white-spotted bars in black color). Plasma levels of methylglyoxal in patients with septic shock are presented at sepsis onset (S/T0) and 24 hours (S/T1) later. Plasma methylglyoxal measurements in postoperative controls are presented immediately after the end of the surgical procedure (P/T1) and 24 hours afterwards (P/T2). Data in bar charts are presented as medians and 95% CIs. With regard to symbolism and higher orders of significance: ***P <0.001.

**Figure 2:**
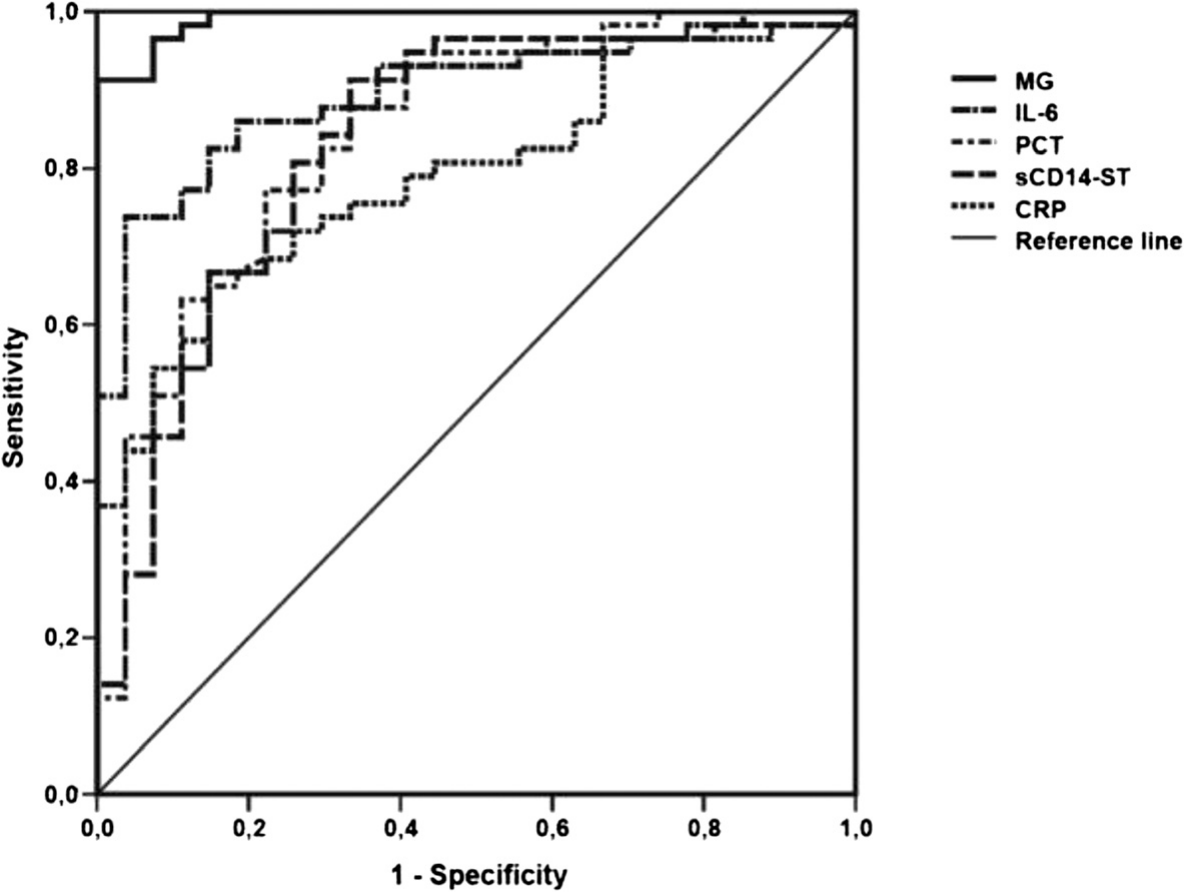
**Diagnostic value of methylglyoxal (MG), C-reactive protein (CRP), procalcitonin (PCT), interleukin-6 (IL-6), and soluble CD14 subtype (sCD14-ST) for identification of patients with sepsis.** Receiver operating characteristic curves for plasma levels of MG (continuous line), IL-6 (tight alternating dashed line), CRP (short dashed line), PCT (wide alternating dashed line), and sCD14-ST (long dashed line) in patients with septic shock at sepsis onset (S/T0, n = 60) and postoperative controls 24 hours after major abdominal surgery (P/T2, n = 30).

In patients with septic shock (n = 60), plasma concentrations of MG were significantly increased in comparison with healthy volunteers (n = 30) (Figure 1a) and compared with postoperative controls (n = 30) (Figure 1b). Similar observations could be made for other routine infection and inflammation markers such as C-reactive protein (CRP), procalcitonin (PCT), IL-6, and sCD14-ST (Table 3 and Additional file 2: Table S2). However, MG proved to be superior for the identification of patients with septic shock (S/T0 versus P/T2) as assessed by area under the curve (AUC) comparisons of the related ROC curves (Figure 2 and Table 4). Besides, plasma levels of MG were shown to be independent of the septic focus (lung, gastrointestinal tract, genitourinary tract, and so on) as well as the underlying pathogen (Gram-positive isolates, Gram-negative isolates, both, and suspected infection without any microbiological finding) (data not shown).

**Table 3:** **Plasma levels of C-reactive protein, procalcitonin, interleukin-6, soluble CD14 subtype, blood glucose, and total antioxidant capacity in the volunteer (V/T0), postoperative (P/T0, P/T1, P/T2), and septic (S/T0, S/T1) groups**

**Parameters**	**Volunteer group (n = 30)**	**Postoperative group (n = 30)**						**Septic group (n = 60)**			
	**V/T0**	**P/T0**		**P/T1**		**P/T2**		**S/T0**		**S/T1**	
CRP, mg/L	2.0	4.3	***	4.3	***	110.2	***	169.2	***	190.4	***
PCT, μg/L	0.06	0.07	ns	0.10	**	0.82	***	4.63	***	7.17	***
IL-6, pg/mL	0.0	0.0	**	215.3	***	132.5	***	500.5	***	337.6	***
sCD14-ST, pg/mL	123.0	208.5	***	711.0	***	680.0	***	1817.5	***	2309.0	***
Blood glucose, mg/dL	85.0	102.5	**	140.0	***	116.0	***	128.5	***	139.0	***
TAC, mM	0.02	0.06	***	0.10	***	0.12	***	0.15	***	0.13	***

Data are presented as medians. Results of pairwise comparisons refer to intergroup comparisons of each presented time point in the postoperative (P/T0, P/T1, P/T2) and septic (S/T0, S/T1) groups with the volunteer group (V/T0). With regard to symbolism and higher orders of significance: *P <0.05; **P <0.01; ***P <0.001; ns, not statistically significant. The central lab of the University Hospital of Heidelberg provides precise values for C-reactive protein (CRP) beginning from 2 mg/L. Lower CRP values are simply denoted less than 2.0 mg/L. For statistical analyses, all values less than 2.0 mg/L were put on a level of 2.0 mg/L. Precise values for PCT are available beginning from 0.05 μg/L. Lower PCT values are simply denoted less than 0.05 μg/L. For statistical analyses, all values less than 0.05 μg/L were put on a level of 0.05 μg/L. IL-6, interleukin-6; PCT, procalcitonin; sCD14-ST, soluble CD14 subtype; TAC, total antioxidant capacity.

**Table 4:** **Diagnostic value of methylglyoxal, C-reactive protein, procalcitonin, interleukin-6, and soluble CD14 subtype for identification of patients with sepsis**

**Parameter**	**AUC (S/T0 vs. P/T2)**	**Cutoff**	**Sensitivity**	**1-Specificity**	**Results of pairwise comparisons**
MG, nM	0.993	281.1	0.912	0.000	
CRP, mg/L	0.791	152.8	0.667	0.148	<0.001***
PCT, μg/L	0.844	1.46	0.772	0.222	0.002**
IL-6, pg/mL	0.898	401.0	0.737	0.037	0.007**
sCD14-ST, pg/mL	0.832	825	0.912	0.333	<0.001***

Receiver operating characteristic (ROC) analysis was performed with suitable parameters in order to create cutoff values to determine the diagnostic value of each parameter with regard to the diagnosis of sepsis (S/T0 versus P/T2). Area under the curve (AUC) comparisons of the related ROC curves refer to pairwise comparisons of each parameter with MG. With regard to symbolism and higher orders of significance: **P <0.01; ***P <0.001. CRP, C-reactive protein; IL-6, interleukin-6; MG, methylglyoxal; ns, not statistically significant; PCT, procalcitonin; sCD14-ST, soluble CD14 subtype.

### Methylglyoxal**-**derived carbonyl stress in human sepsis – Relevance for the progression of the disease?

**Figure 3:**
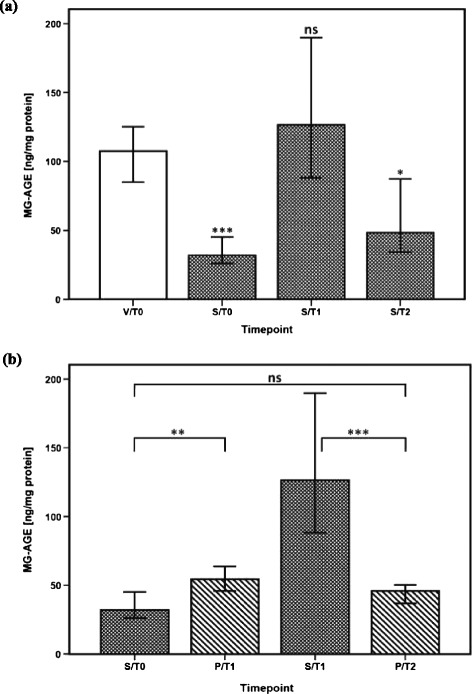
**Methylglyoxal-derived advanced glycation end product (MG-AGE) formation in different inflammatory settings in humans. (a)** Comparisons of plasmatic MG-AGE formation in healthy volunteers (n = 30, white bar) and patients with septic shock (n = 60, white-spotted bars in black color). Plasma levels of MG-AGEs in patients with septic shock are presented at sepsis onset (S/T0) and 24 hours (S/T1) and 4 days (S/T2) later. Plasma measurements of MG-AGEs in healthy volunteers were performed once (V/T0). Data in bar charts are presented as medians and 95% confidence intervals (CIs). With regard to symbolism and higher orders of significance: *P <0.05; ***P <0.001. Asterisks refer to pairwise comparisons between healthy volunteers and patients with septic shock at the presented time points. **(b)** Comparisons of plasma MG-AGE formation in postoperative controls following major abdominal surgery (n = 30, white-striped bars in black color) and patients with septic shock (n = 60, white-spotted bars in black color). Plasma levels of MG-AGEs in patients with septic shock are presented at sepsis onset (S/T0) and 24 hours (S/T1) later. Plasma measurements of MG-AGEs in postoperative controls are presented immediately after the end of the surgical procedure (P/T1) and 24 hours afterwards (P/T2). Data in bar charts are presented as medians and 95% CIs. With regard to symbolism and higher orders of significance: **P <0.01; ***P <0.001. ns, not statistically significant.

Within a 28-day and 90-day observation period, plasma levels of MG at sepsis onset were significantly higher in non-survivors than in the corresponding survivors (Additional file 3: Figure S1a and Additional file 4: Figure S2a). Accordingly, plasma levels of MG proved to be an early predictor for survival in patients with septic shock (sepsis onset: ROC-AUC 0.710; cutoff: 591.8 nM → sensitivity: 0.700; 1-specificity: 0.300 for 28-day survival; ROC-AUC 0.686; cutoff: 568.4 nM → sensitivity: 0.655; 1-specificity: 0.308 for 90-day survival) (Additional file 3: Figure S1b and Additional file 4: Figure S2b). In contrast, plasma levels of MG did not differ significantly between patients with sepsis-associated organ failures—for example, acute liver failure, acute renal failure, and acute respiratory distress syndrome—in comparison with those septic patients with unimpaired organ function (data not shown). One main effect of MG is the post-translational modification of proteins to form AGEs. Accordingly, the levels of plasma MG-AGEs reached their maximum at 24 hours after sepsis onset in patients who developed septic shock. However, plasma levels of MG-AGEs failed to be significantly higher in patients with septic shock compared with healthy volunteers. In contrast, plasma levels of MG-AGEs were significantly elevated in patients with septic shock in comparison with postoperative controls at 24 hours (Figure 3a and 3b).

### Methylglyoxal**-**derived carbonyl stress in human sepsis - a consequence of increased methylglyoxal formation?

Blood sugar levels and the corresponding rate of glycolysis were hypothesized to have a relevant influence on plasmatic MG levels. Accordingly, patients with septic shock and postoperative controls showed significantly increased blood sugar levels over the entire study period in comparison with healthy volunteers (Table 3). However, there was only a weak correlation between plasma levels of MG and blood sugar levels (r = 0.139) in all study groups, implicating the contribution of other factors (for example, oxidative stress) to the measured increased plasma levels of MG than solely hyperglycemia. Healthy volunteers were shown to experience a low grade of oxidative stress, as indirectly assessed by plasma TAC. In contrast, postoperative controls already showed increased levels of oxidative stress prior to surgery (P/T0)_,_ which further increased over time after the surgical procedure (P/T1, P/T2). Compared with both control groups, patients with septic shock were found to have the highest level of oxidative stress within 24 hours after sepsis onset (S/T0, S/T1) (Table 3) and plasma TAC was found to positively correlate with MG plasma levels (r = 0.348) in all study groups.

### Methylglyoxal**-**derived carbonyl stress in human sepsis - a consequence of inhibited methylglyoxal detoxification?

**Figure 4:**
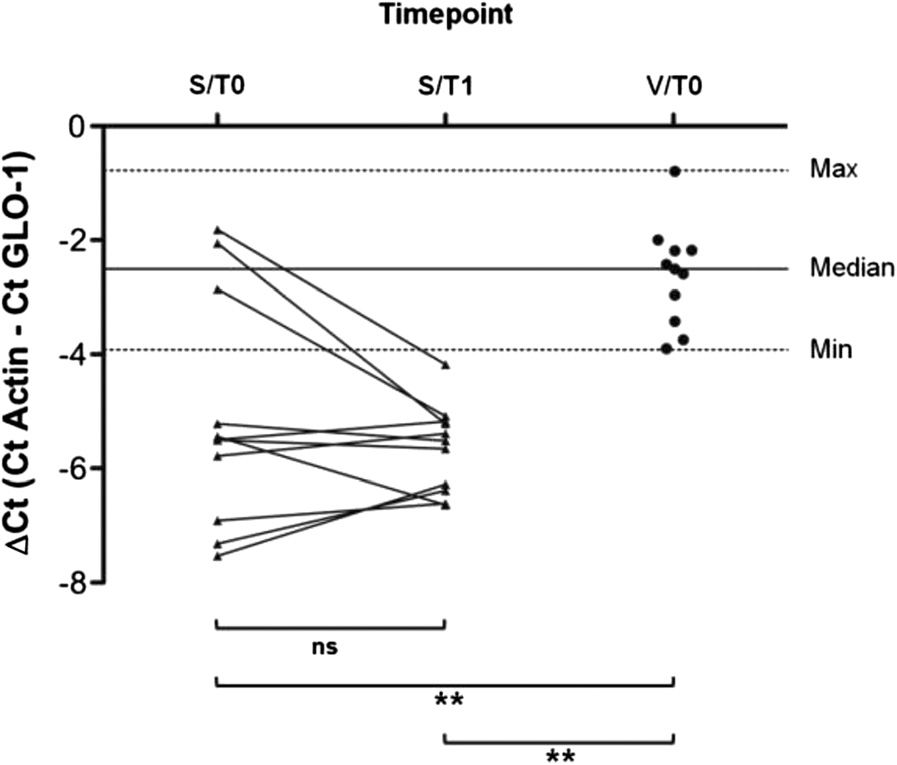
**Methylglyoxal-detoxifying capacity in different inflammatory settings in humans.** Glyoxalase-1 (GLO-1) expression in peripheral blood mononuclear cells of patients with septic shock compared with healthy volunteers is shown. Samples from 11 patients with septic shock—at onset (S/T0) and 24 hours (S/T1) later—and 11 healthy volunteers (V/T0) were analyzed by quantitative reverse transcription-polymerase chain reaction. Dotted horizontal lines represent the minimum and maximum change in cycle threshold (∆Ct) values of healthy volunteers, and solid lines shows the median accordingly. With regard to symbolism and higher orders of significance: **P <0.01. ns, not statistically significant.

In comparison with healthy volunteers, expression of GLO-1, the key enzyme of the MG detoxification pathway, was shown to be significantly reduced in patients with septic shock within the first 24 hours after sepsis onset (S/T0, S/T1) (Figure 4).

## Discussion

In this study, MG was identified as a marker for monitoring the onset, development, and remission of sepsis. It was found to be more useful than routine diagnostic markers, such as CRP, PCT, IL-6, and sCD14-ST. This finding is of great interest, as it has been suggested that the currently used biomarkers for sepsis do not have either the specificity or sensitivity to be routinely used in clinical practice [17].

The major source of endogenous MG is from the non-enzymatic degradation of the triosephosphate intermediates within glycolysis. Under conditions of increased glycolytic flux, such as hyperglycemia (a symptom often observed in patients with sepsis), it would be safe to assume that there should be an increase in MG production. In this study, blood sugar levels were significantly elevated in patients with septic shock. Measured plasma MG in these patients was also elevated, but only a weak correlation with blood glucose levels could be shown. This would suggest that there are other factors contributing to the measured increased plasma levels of MG than only hyperglycemia.

In this study, patients with septic shock characteristically have an increased state of oxidative stress and this correlates positively with plasma MG levels, suggesting that a relationship exists between these two forms of metabolic stress. GLO-1, the major pathway for detoxification of MG, is a glutathione (GSH)-dependent enzyme [18]. Previous studies have shown that under conditions of oxidative stress, both GSH and nicotinamide-adenine-dinucleotide-phosphate-hydrate (NADPH) are depleted, which in turn decreases the *in situ* activity of GLO-1 [19], thereby increasing the concentration of MG. The loss of essential cofactors in a situation of increased oxidative stress could therefore be one explanation for the increased plasma MG observed in the patients with sepsis. However, it was also found that patients with sepsis syndrome had a significant reduction in the expression of mononuclear GLO-1, suggesting that patients with sepsis are more susceptible to the accumulation of MG through the loss of the GLO-1 rather than the loss of an essential cofactor. It has been proposed that the engagement of the receptor for advanced glycation end products (RAGE) by inflammatory mediators, such as carboxylmethyl-lysine (CML) and high-mobility group box protein-1 (HMGB1), can reduce expression of GLO-1 [20]. In this study, the cell-surface expression of RAGE was increased in the monocytes of patients with septic shock; however, there was also a significant reduction in the plasma concentration of the classic RAGE ligands (data not shown). It has also been proposed that hyperglycemic conditions, such as those observed in diabetes, can directly reduce GLO-1 activity. However, the underlying mechanism for this effect remains unclear [21].

One of the primary effects of elevated MG is the post-translational modification of proteins to form AGEs [22,23]. This study could show that the concentration of MG-AGEs in plasma paralleled the levels of MG and that the highest concentrations were observed 24 hours after the onset of sepsis. This may simply reflect a cause-and-effect relationship in the plasma; however, it may also indicate an increased turnover of MG-modified or damaged proteins within the tissue [6]. Such modified proteins are degraded by cellular proteolysis, releasing not only modified peptides but also the modified amino acid, which is eventually excreted in the urine [6]. Several studies have shown that MG-derived hydroimidazolone (MG-H1) [24], the adduct formed from MG modification of arginine, is the major quantitative modified adduct excreted in humans and rats [25,26]. Increased excretion of this modified product, particularly in diabetes mellitus, is associated with the development and progression of complications, such as neuropathy, nephropathy, and retinopathy [9,27-30]. It has also been shown that MG-AGEs, specifically MG-H1, can interact with RAGE. Monocytes can bind, internalize, and degrade albumin which has been minimally modified by MG, leading to synthesis and secretion of pro-inflammatory mediators such as IL-1β, macrophage colony-stimulating factor, and tumor necrosis factor-alpha. It has been suggested that this binding is mediated by RAGE [31-35], making it possible that the high circulating levels of MG-AGE proteins found in patients with sepsis could activate circulating monocytes by ligating RAGE. This may result in the downregulation of GLO-1 and the induction of the pro-inflammatory phenotype observed in patients with septic shock. Further *in vitro* studies using MG-modified proteins, modified to a similar extent as those observed in patients with sepsis, are required to confirm this observation.

The findings of increased MG and MG protein damage in sepsis suggest that MG metabolism is an important and generally overlooked biochemical pathway for the induction of cellular dysfunction or inflammation (or both) in sepsis. Furthermore, prevention of MG overload might represent a new therapeutic option in sepsis. Unfortunately, there are currently no MG scavengers approved for clinical use. As in diabetes, tight blood glucose control in patients with sepsis may offer a strategy for reducing the amount of MG accumulation. Accordingly, recent clinical guidelines for the treatment of sepsis have recommended that blood glucose levels should be maintained at not more than 180 mg/dL [36]. An alternative treatment strategy could be to increase GLO-1 activity. It has been shown *in vitro* that GLO-1 transcription is regulated by the antioxidant transcription factor nuclear factor (erythroid-derived 2)-like 2 (Nrf2) [37]. Activation of this transcription factor by isothiocyanates, such as sulphoraphane, can lead to increased GLO-1 activity and decreased MG and MG-derived AGEs [38]. Such compounds are found in high abundance in cruciferous vegetables. However, the effect of such treatment *in vivo* remains unknown and requires further investigation to determine whether it would be effective in acute illnesses such as sepsis.

Further investigations are required to determine whether the diagnostic and prognostic value of MG can be confirmed and validated in a large study cohort. Moreover, to link the clinical observations of our study to functional implications, it needs to be evaluated *in vitro*, whether MG modifications are responsible for the cellular dysfunction or inflammation (or both) observed in sepsis. Critical to this process would be to identify the cellular targets involved in this modification. It could already be shown that mitochondrial proteins are particularly susceptible to modification by MG [39]. The ELISA-based method used to detect MG-AGEs in this study is unable to differentiate whether the MG-AGEs measured are complete proteins, peptides, or the free amino acids. In future studies, the measurement of both MG and MG-AGEs should be performed by using the gold-standard technique of stable isotope dilution, liquid chromatography tandem mass spectroscopy, which could provide robust identification, particularly with respect to the proteins or amino acids (or both) modified by MG [26,40].

## Conclusions

**Figure 5:**
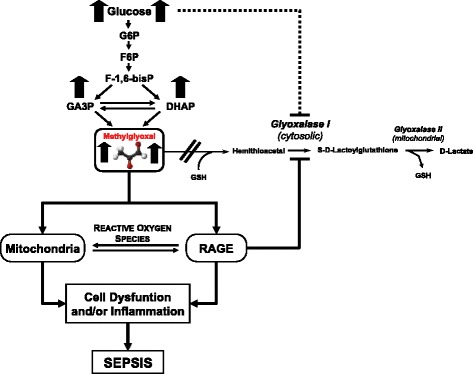
**Mechanism of methylglyoxal and methylglyoxal-derived advanced glycation end products driven cell dysfunction or inflammation or both in human sepsis.** DHAP, dihydroxyacetone phosphate; F-1,6-bisP, fructose-1,6-bisphosphate; F6P, fructose 6-phosphate; G6P, glucose 6-phosphate; GA3P, glyceraldehyde 3-phosphate; GSH, glutathione; RAGE, receptor for advanced glycation end products.

The role of RCS, such as MG, has generally been overlooked in the context of the sepsis syndrome. In this study, MG was identified as a better marker for the identification of patients with sepsis in comparison with routine diagnostic markers. Furthermore, MG was shown to be an early predictor for survival in patients with septic shock. It is hypothesized that in sepsis, increased glucose metabolism resulting from acute hyperglycemia leads to increased production of MG and MG-AGEs. Activation of RAGE by MG-AGEs leads to the downregulation of GLO-1, and a positive feedback loop then leads to further accumulation of MG as well as the transcription of proinflammatory genes. The modification of different (mitochondrial) proteins by MG could lead to increased oxidative stress, further enhancing the MG-activated pathways of cellular dysfunction and inflammation (Figure 5). Further studies are required to determine the extent of MG modification in sepsis and its relative importance to the progression and remission of the condition and whether targeting this pathway would be of therapeutic benefit to the patient.

## Key messages

- MG was identified as a better biomarker than the established routine markers (for example, CRP, PCT, IL-6, and sCD14-ST) for the identification of patients with sepsis.
- MG was shown to be an early predictor for survival in patients with septic shock.
- The implementation of MG measurements in routine diagnostics of patients with suspected sepsis should therefore be taken into consideration.
- The relative importance of MG to the progression and remission of septicemia, and whether targeting MG-derived carbonyl stress would be of therapeutic benefit to the patient, needs to be further evaluated.

## Additional files

Additional file 1: Table S1. Definitions of infection [13]. Additional file 2: Table S2. Results of pairwise comparisons of plasma levels of C-reactive protein, procalcitonin, interleukin-6, soluble CD14 subtype, blood glucose, and total antioxidant capacity in the postoperative (P/T1, P/T2) and septic (S/T0, S/T1) groups. Additional file 3: Figure S1. Prediction of short-term survival in patients with septic shock based on methylglyoxal plasma levels. **(a)** Comparison of plasma methylglyoxal measurements in patients with septic shock who ultimately did and did not survive within the 28-day observation period. Plasma levels of methylglyoxal in surviving and non-surviving patients with septic shock are presented at sepsis onset (S/T0), 24 hours (S/T1), 4 days (S/T2), 7 days (S/T3), 14 days (S/T4), and 28 days (S/T5). Data in bar charts are presented as medians and 95% confidence intervals (CIs). With regard to symbolism and higher orders of significance: ***P* <0.01. **(b)** Receiver operating characteristic curve for plasma levels of methylglyoxal (continuous line) at sepsis onset in patients with septic shock who ultimately did and did not survive within the 28 days observation period. Additional file 4: Figure S2. Prediction of medium-term survival in patients with septic shock based on methylglyoxal plasma levels. **(a)** Comparison of plasma methylglyoxal measurements in patients with septic shock who ultimately did and did not survive within the 90-day observation period. Plasma levels of methylglyoxal in surviving and non-surviving patients with septic shock are presented at sepsis onset (S/T0), 24 hours (S/T1), 4 days (S/T2), 7 days (S/T3), 14 days (S/T4), and 28 days (S/T5). Data in bar charts are presented as medians and 95% confidence intervals (CIs). With regard to symbolism and higher orders of significance: **P* <0.05. **(b)** Receiver operating characteristic curve for plasma levels of methylglyoxal (continuous line) at sepsis onset in patients with septic shock who ultimately did and did not survive within the 90-day observation period.

## Abbreviations

AGE: advanced glycation end product
AUC: area under the curve
CRP: C-reactive protein
Ct: cycle threshold
ELISA: enzyme-linked immunosorbent assay
GLO-1: glyoxalase-I
GSH: glutathione
ICU: intensive care unit
IL: interleukin
MG: methylglyoxal
MG-AGE: methylglyoxal-derived advanced glycation end products
MG-H1: methylglyoxal-derived hydroimidazolone
PBMC: peripheral blood mononuclear cell
PCR: polymerase chain reaction
PCT: procalcitonin
RAGE: receptor for advanced glycation end products
RCS: reactive carbonyl species
ROC: receiver operating characteristic
ROS: reactive oxygen species
sCD14-ST: soluble CD14 subtype
TAC: total antioxidant capacity
