# Supplementary material for: Methylglyoxal as a new biomarker in patients with septic shock: an observational clinical study
Source: Crit Care. 2014 Dec 12;18(6):683. doi: 10.1186/s13054-014-0683-x (PMC4301657; doi:10.1186/s13054-014-0683-x)
Supplement: Additional file 1: Table S1. — Definitions of infection [13]. [file 13054_2014_683_MOESM1_ESM.doc]

| **Organ** | **Manifestation** | **Infection criteria** |
| --- | --- | --- |
| Lung | Pneumonia | - radiographic evidence of infiltrate - microbiological or serological confirmation - clinical signs: cough, sputum, impaired oxygenation, breath-related pain, breath sounds |
| Gastrointestinal tract | Peritonitis | - radiographic evidence: free air or liquid - microbiological evidence - clinical signs: pain, peritonism, nausea and vomiting, fever |
| Genitourinary tract | Pyelonephritis | - radiological evidence - microbiological evidence - clinical signs: dysuria, pollakisuria, hematuria, flank pain, fever |
| Skin and soft tissue | Cellulitis / necrotizing infections | - microbiological evidence - local signs: redness, swelling, pus, lymphangitis, lympadenopathy, necrosis, pain |
| Blood | Bloodstream infection | - positive blood culture - local signs: erythrema and swelling at entry site of catheter, pus |

**Additional file 1: Table S1.** Definitions of infection [13].
