# Supplementary material for: Methylglyoxal as a new biomarker in patients with septic shock: an observational clinical study
Source: Crit Care. 2014 Dec 12;18(6):683. doi: 10.1186/s13054-014-0683-x (PMC4301657; doi:10.1186/s13054-014-0683-x)
Supplement: Additional file 2: Table S2. — Results of pairwise comparisons of plasma levels of C-reactive protein, procalcitonin, interleukin-6, soluble CD14 subtype, blood glucose, and total antioxidant capacity in the postoperative (P/T1, P/T2) and septic (S/T0, S/T1) groups. [file 13054_2014_683_MOESM2_ESM.doc]

**Additional file 2: Table S2.** Results of pairwise comparisons of plasma levels of C-reactive protein, procalcitonin, interleukin-6, soluble CD14 subtype, blood glucose and total antioxidant capacity in the postoperative (P/T1, P/T2) and septic groups (S/T0, S/T1).

| **Parameters** | **S/T0 vs. P/T1** | **S/T1 vs. P/T2** |
| --- | --- | --- |
| CRP | ******* | ******* |
| PCT | ******* | ******* |
| IL-6 | ******* | ******* |
| sCD14-ST | ******* | ******* |
| Blood glucose | ns | ****** |
| TAC | ******* | ***** |
| With regard to symbolism and higher orders of significance: p < 0.001: ***; p<0.01: **; p < 0.05*; ns: not statistically significant.  Abbreviations: CRP, C-reactive protein; PCT, procalcitonin; IL-6, interleukin-6; sCD14-ST, soluble CD14 subtype; TAC, total antioxidant capacity | | |
