# Supplementary figures and images for: Methylglyoxal as a new biomarker in patients with septic shock: an observational clinical study
Source: Crit Care. 2014 Dec 12;18(6):683. doi: 10.1186/s13054-014-0683-x (PMC4301657; doi:10.1186/s13054-014-0683-x)

**Additional file 3: Figure S1.**

**(a)**

**
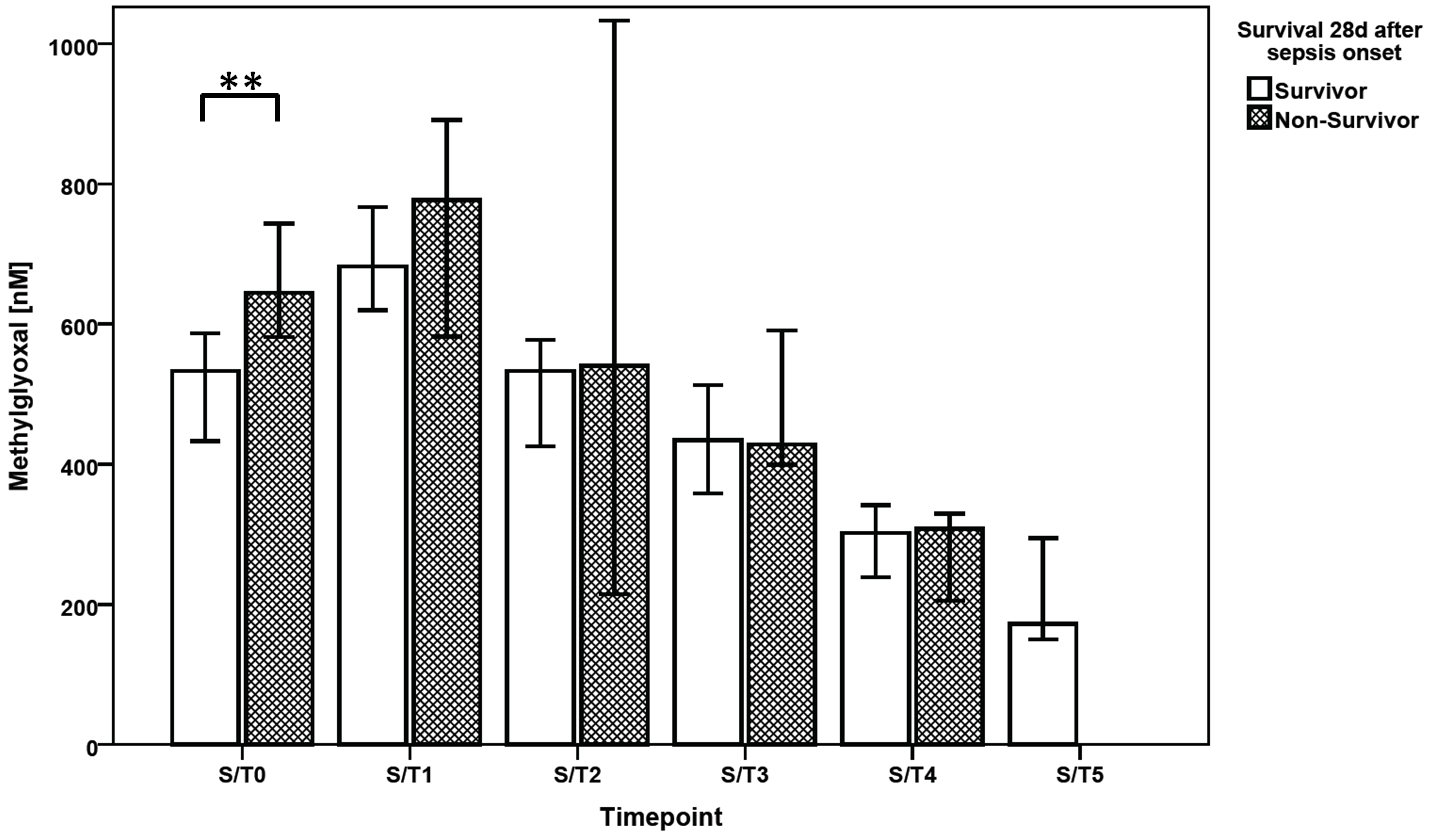
**

**(b)**

**
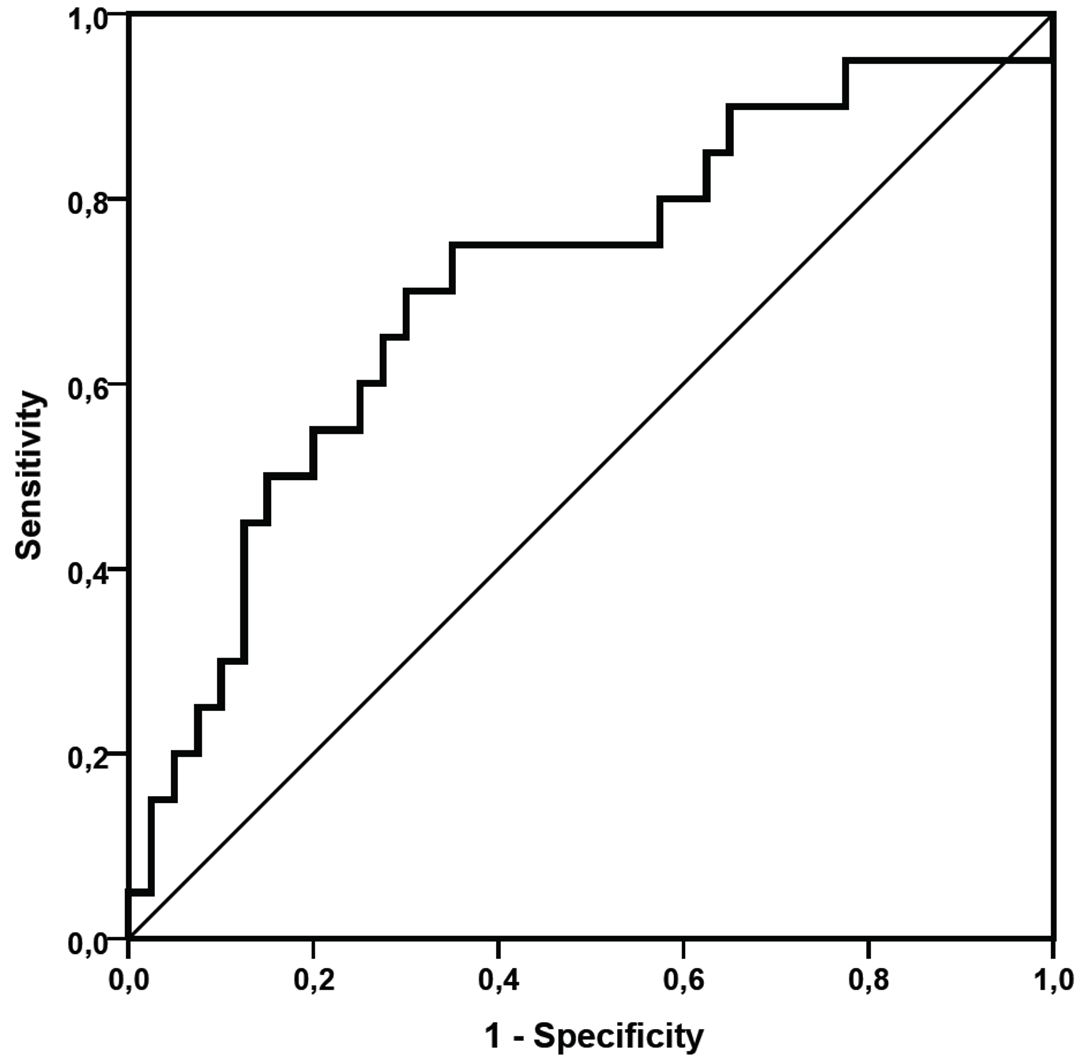
**

Supplement: Additional file 3: Figure S1. — Prediction of short-term survival in patients with septic shock based on methylglyoxal plasma levels. (a) Comparison of plasma methylglyoxal measurements in patients with septic shock who ultimately did and did not survive within the 28-day observation period. Plasma levels of methylglyoxal in surviving and non-surviving patients with septic shock are presented at sepsis onset (S/T0), 24 hours (S/T1), 4 days (S/T2), 7 days (S/T3), 14 days (S/T4), and 28 days (S/T5). Data in bar charts are presented as medians and 95% confidence intervals (CIs). With regard to symbolism and higher orders of significance: **P <0.01. (b) Receiver operating characteristic curve for plasma levels of methylglyoxal (continuous line) at sepsis onset in patients with septic shock who ultimately did and did not survive within the 28 days observation period. [file 13054_2014_683_MOESM3_ESM.doc]

**Additional file 4: Figure S2.**


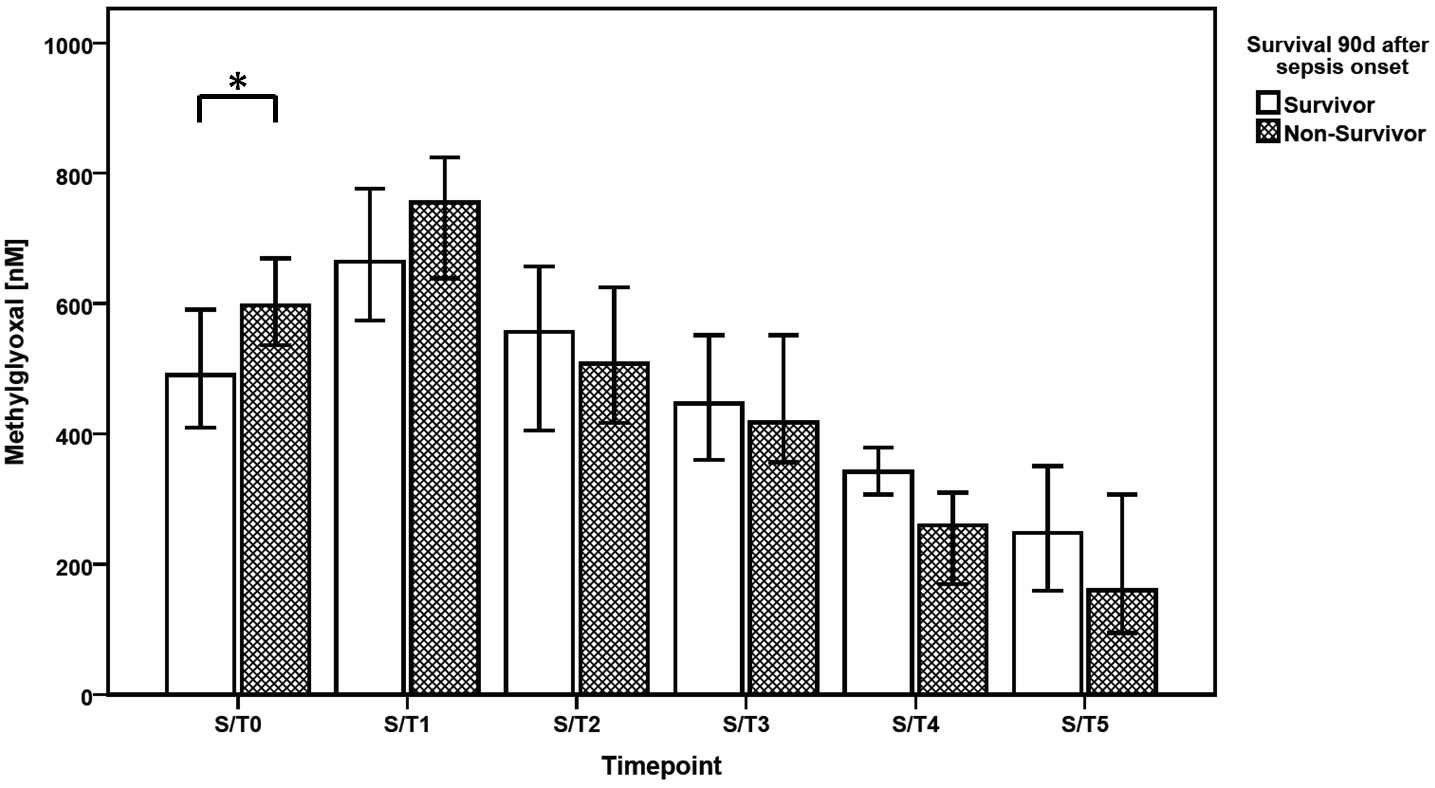
**(a)**


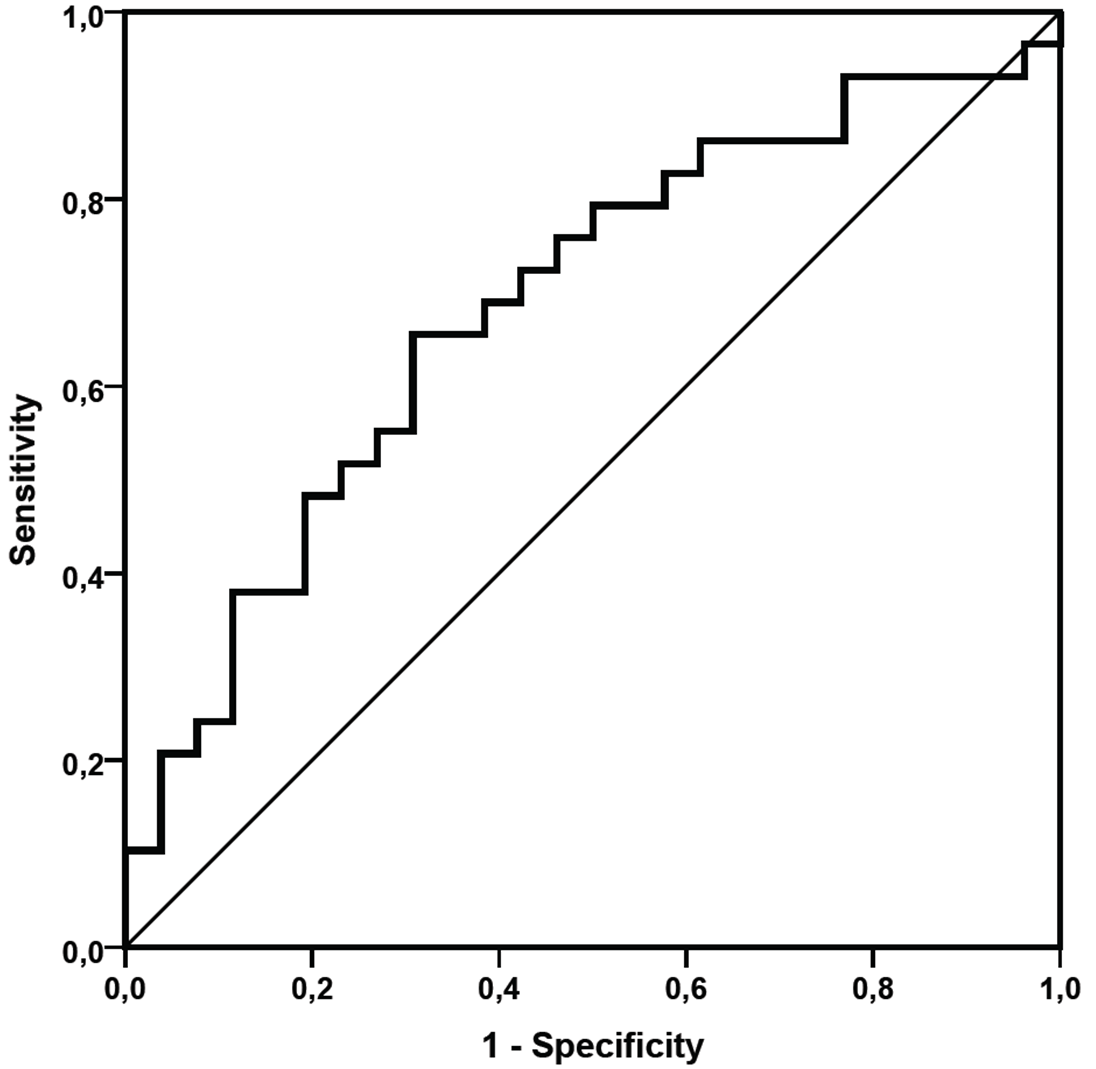
**(b)**

Supplement: Additional file 4: Figure S2. — Prediction of medium-term survival in patients with septic shock based on methylglyoxal plasma levels. (a) Comparison of plasma methylglyoxal measurements in patients with septic shock who ultimately did and did not survive within the 90-day observation period. Plasma levels of methylglyoxal in surviving and non-surviving patients with septic shock are presented at sepsis onset (S/T0), 24 hours (S/T1), 4 days (S/T2), 7 days (S/T3), 14 days (S/T4), and 28 days (S/T5). Data in bar charts are presented as medians and 95% confidence intervals (CIs). With regard to symbolism and higher orders of significance: *P <0.05. (b) Receiver operating characteristic curve for plasma levels of methylglyoxal (continuous line) at sepsis onset in patients with septic shock who ultimately did and did not survive within the 90-day observation period. [file 13054_2014_683_MOESM4_ESM.doc]
